# The addition of dydrogesterone improves the outcomes of pregnant women with low progesterone levels when receiving vaginal progesterone alone as luteal support in HRT‐FET cycles

**DOI:** 10.1002/rmb2.12511

**Published:** 2023-03-22

**Authors:** Rena Toriumi, Michiharu Horikawa, Chie Sato, Nagisa Shimamura, Rena Ishii, Michiru Terashima, Michiko Hamada, Naoyuki Tachibana, Yuji Taketani

**Affiliations:** ^1^ Women's Clinic Oizumigakuen, Lenia Medical Corporation Tokyo Japan; ^2^ Artemis Women's Hospital, Lenia Medical Corporation Tokyo Japan

**Keywords:** ART HRT‐FRT cycle, dydrogesterone, luteal support, serum progesterone, vaginal progesterone

## Abstract

**Purpose:**

Vaginal progesterone (VP) alone has been used as luteal support (LS) in HRT‐FET cycles without measuring serum progesterone concentrations (SPC) because it can achieve adequate intrauterine progesterone levels. However, several reports showed that the co‐administration of progestin produced better outcomes than VP alone. We tried to address this discrepancy, focusing on SPC.

**Methods:**

VP was given to 180 women undergoing HRT‐FET. We measured SPC when pregnancy was diagnosed on day 14 of LS. We compared assisted reproductive technology outcomes between VP alone versus VP + dydrogesterone (D).

**Results:**

When using VP alone, average SPC in the miscarriage cases (9.6 ng/mL) were significantly lower compared with the ongoing pregnancy (OP) cases (14.7 ng/mL). The cut‐off value for progesterone, 10.7 ng/mL, was a good predictor for the subsequent course of the pregnancy. Of 76 women receiving D ± VP from the start of LS and achieving a pregnancy, the numbers of OP were 44 (84.6%) in SPC ≥ 10.7 ng/mL and 20 (83.3%) in SPC ≤ 10.7 ng/mL with no significant difference.

**Conclusion:**

VP alone resulted in lower SPC in some pregnant women in HRT‐FET cycles and exhibited a lower OP rate. The co‐administration of D improved an OP rate of low progesterone cases to the level comparable with non‐low progesterone cases.

## 1 INTRODUCTION

Frozen–thawed embryo transfer (FET) has been increasingly preferred in assisted reproductive technology (ART). This method has the advantages of reducing the risk of ovarian hyperstimulation syndrome and the utilization of all eggs. In addition, performing FET in combination with hormone replacement therapy (HRT) makes it possible to schedule the day of FET to the patient's convenience by adjusting the day when luteal support is started.

Currently, there are no evidence‐based guidelines for the HRT. Due to the lack of a functional corpus luteum, sex steroid hormones required for the endometrium to acquire egg receptivity depend exclusively on exogenous synthetic hormones. Progesterone (P) plays a crucial role together with estradiol in the endometrium to exert its physiological roles. In a physiological setting, the blood concentrations of P in the luteal phase fluctuate daily.^1^ Moreover, P concentrations exhibit circadian rhythms in a menstrual cycle‐dependent manner.^2^ It is thought that such finely regulated secretory dynamics of P could be of critical relevance to the establishment of pregnancy. However, the replacement of P has been empirically conducted, yielding acceptable, if not excellent, ART outcomes.

There are various routes of P/progestins administration in ART. The development of vaginal preparations has increased treatment compliance. Vaginal administration of P has the first uterine pass effect; thus, significantly higher endometrial P levels are obtained compared with systemic administration.^3^ Hence, vaginal preparations have been thought to be used alone as luteal support without considering P concentrations in the blood; therefore, the transvaginal route has become the mainstream luteal support.^4^, ^5^

However, as the use of vaginal preparations has become widespread, many lines of investigation have emerged that suggest that the combined use of vaginal P with other routes of P/progestins could improve the outcomes of ART using HRT‐FET over vaginal P alone, thus questioning the validity of the notion that vaginal P alone is sufficient for luteal support.^6^, ^7^, ^8^, ^9^ In this regard, it has been suggested that serum P levels do not rise sufficiently in some women receiving vaginal P, leading to poor ART outcomes.^6^, ^7^, ^8^

The present study was concluded to determine whether the outcomes of the pregnancies by ART using HRT‐FET are related to the serum P levels. We further determine whether the outcomes really improve by supplementing progestins, even in cases where serum P levels are actually low. To the best of our knowledge, this is the first study to examine the effect of additional progestin administration on the outcomes of the pregnancies conceived by ART using HRT‐FET while relating the outcomes to serum P levels when a human chorionic gonadotropin (hCG) pregnancy test is positive.

Herein, we demonstrate that when vaginal P alone is used as luteal support in an HRT‐FET cycle, a lack of P actions, which is reflected in lower serum P levels, often occurs. This appears to increase the risk of miscarriage even if pregnancy is achieved. In addition, we further demonstrate that this could be overcome by supplementing synthetic progestin, dydrogesterone with vaginal P.

## 2 MATERIALS AND METHODS

We enrolled women with regular or irregular menstrual cycles who underwent FET in an HRT cycle that resulted in a clinical pregnancy from July 2019 to October 2021 in the Women's Clinic Oizumigakuen, Tokyo, Japan. For egg collection, the following ovarian stimulation protocols were used: clomiphene citrate (Clomid, Fuji Pharma Co., Ltd.), clomiphene citrate + gonadotropin (Folyrmon‐P, Fuji Pharma Co., Ltd.) (hMG‐Fuji, Fuji Pharma Co., Ltd.) (hMG‐ferring, Ferring Pharmaceuticals), gonadotropin + gonadotropin‐releasing hormone (GnRH) agonist, or gonadotropin + GnRH antagonist (Relumina, Asuka Pharmaceutical Co., Ltd.). The main indications for infertility treatment included unexplained infertility, diminished ovarian reserve mainly due to ovarian aging, male infertility, and female infertility, such as endometriosis and tubo‐peritoneal factors. Age was not specified in the inclusion criteria for this study.

Estrogen and progestin were consecutively administered to prepare the endometrium for embryo implantation. The administration of estrogen was started on day 2 of the menstrual cycle every day to proliferate the endometrium and inhibit spontaneous follicle growth. The thickness of the endometrium was measured, and the lack of developing follicles was also confirmed using transvaginal ultrasound, usually on days 12–14 of estradiol administration and the day of embryo transfer. Estrogen was administered as an oral tablets of estradiol valerate (Progynova, Bayer) in increasing doses. HRT was started at the patients' convenience, regardless of the length of time since the preceding treatment. When the endometrial thickness was ≥8 mm, the supplementation of P/progestins was commenced together with estradiol to induce secretory changes in the endometrium.

This study compared the outcomes of pregnancy using three different regimens of luteal support. The specific regimens used in the three studies are described below. In all the studies, vaginal micronized P (Utrogestan, Fuji Pharma Co., Ltd.) inserts (200 mg/capsule; three tablets per day) were administered throughout the entire luteal support period to all women who participated in this study, whereas the mode of the additional P/progestins administration differed between the respective studies described below. Luteal support was continued until the end of 9 weeks of pregnancy as long as the pregnancy continued.

The frozen–thawed embryos used in the present study were blastocysts. We recommended a single embryo transfer, whereas multiple embryos were transferred in limited cases considering embryo quality and maternal age. Frozen–thawed blastocysts were transferred on day 5 of luteal support.

Serum hCG concentration was determined on day 14 of luteal support. When the hCG level was ≥15 IU/mL, the pregnancy test result was considered positive. If the pregnancy test was positive, serum concentrations of P and estradiol were further measured on the day hCG levels were determined.

The following three studies differed only in the mode of administration of P/progestins. We planned studies 2 and 3 based on the results of studies 1 and 2, respectively. Therefore, these studies were necessarily conducted at different times. However, they were performed using common ART procedures, except for the regimen of luteal support; thus, the data of each study were considered to mutually comparable.

### 2.1 Study 1

Study 1 included 103 women who achieved a clinical pregnancy in an HRT‐FET cycle while receiving the vaginal P alone as luteal support from July 2019 to May 2020. Vaginal P was administered until the 9th week of pregnancy as long as the pregnancy continued. The women were divided into two groups: those whom pregnancy progressed uneventfully up to the 9th week of pregnancy (ongoing pregnancy group) and those who had a miscarriage by that time (miscarriage group). We compared the serum hormone levels on day 14 of luteal support between the two groups.

### 2.2 Study 2

We additionally administered dydrogesterone (Duphaston, Mylan) 15 mg three times a day and intramuscular injection of P (Proge Depot, Mochida Pharmaceutical Co., Ltd.) 125 mg weekly together with vaginal P to 27 women with *p* < 10.7 ng/mL from day 14 of luteal support until the end of the 9th week of pregnancy as long as the pregnancy continued. The serum P level of 10.7 ng/mL is the cut‐off value determined using the receiver operating characteristic (ROC) curve based on the Study 1 data and shown to predict the outcomes of subsequent pregnancies well, as stated in the “Results” section.

### 2.3 Study 3

From June 2021 to October 2021, dydrogesterone 15 mg three times a day was co‐administered with the vaginal P to 76 women throughout the entire period of luteal support. We determined serum P concentrations mostly derived from the vaginal P because dydrogesterone and its metabolites do not affect the measurement of P.^10^

**FIGURE 1:**
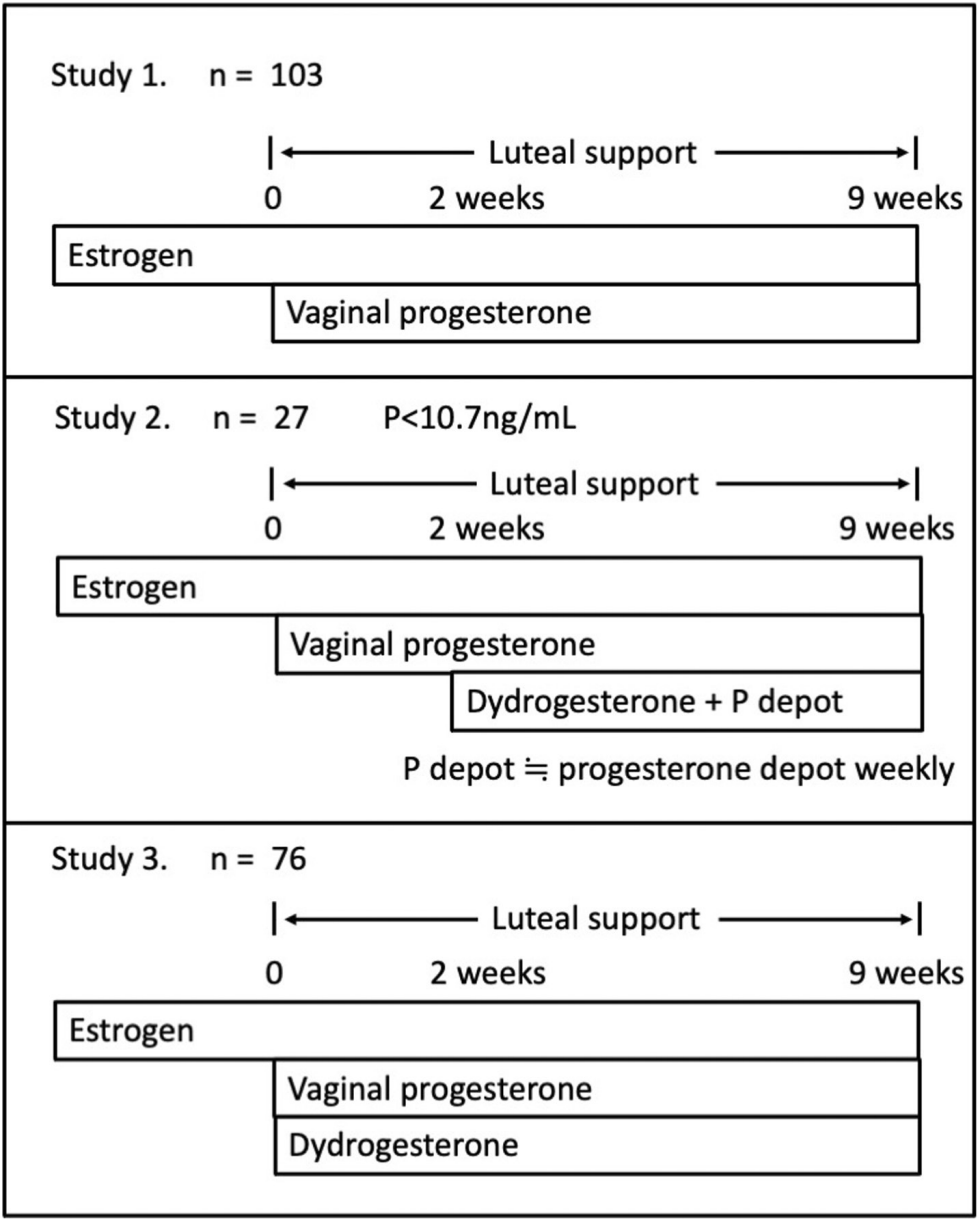
Illustration of the three study designs.

The above three study designs are illustrated in Figure 1.

The data were analyzed by using EZR software (a modified version of R commander).^11^ Statistical significance was set at *p* < 0.05. The study was approved by the institutional ethics committee of Lenia Medical Corporation. All the patients provided informed consent to participate in this study.

## 3 RESULTS

### 3.1 Study 1

First, the overall clinical pregnancy rate was 45.6%. We investigated whether the serum P level on day 14 of luteal support was related to the outcomes of 103 pregnant women who used only vaginal P inserts as a P replacement from the beginning to the end. There were 80 ongoing pregnancies (77.6%) and 23 miscarriages before the end of 9 weeks of pregnancy (22.3%). There was no difference between the two groups in the endometrium thickness on the day luteal support was started and the day of embryo transfer.

**FIGURE 2:**
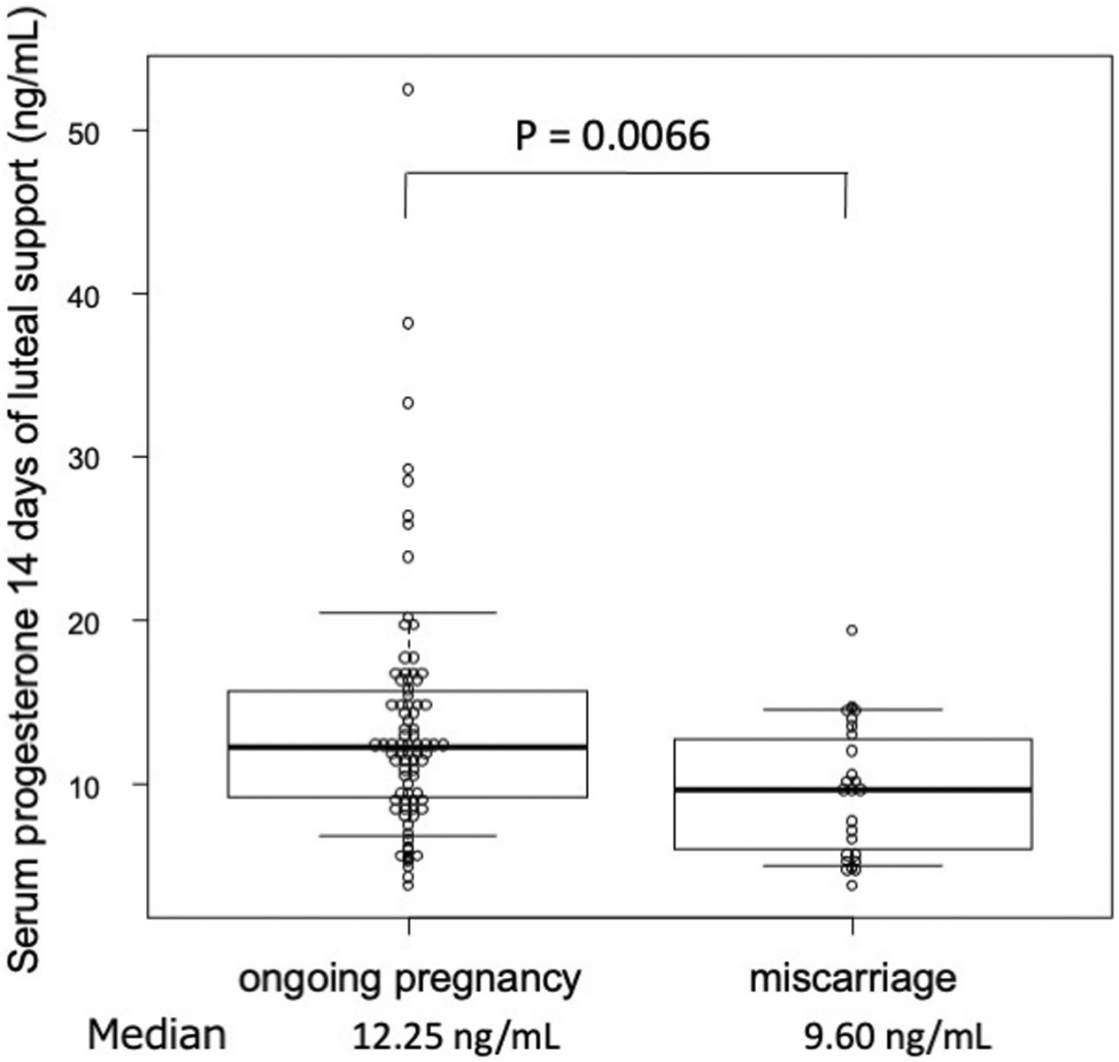
The distribution of serum progesterone levels: a comparison between ongoing pregnancy cases vs. miscarriage cases.

The average P level in the ongoing pregnancy group (14.7 ng/mL) was significantly higher than that in the miscarriage group (9.6 ng/mL). This can be interpreted as a result of the difference in the absorption rate of P through the vaginal wall between the two groups. The average estradiol levels did not differ significantly between the two groups (Table 1). In HRT‐FET cycles, these hormones in the serum on day 14 of luteal support are mostly derived from extrinsic synthetic hormones unrelated to gonadotropin stimulation or embryonic developmental status. The serum hCG levels were significantly higher in the ongoing pregnancy group than that in the miscarriage group. The distribution of serum P concentrations in both groups is shown in Figure 2. There was a significant difference in the distribution of serum P levels between the two groups. Notably, there was a 10‐fold or greater difference in serum P concentration despite using the same dose of vaginal P inserts.

**TABLE 1:** Hormonal profiles and clinical data of Study 1.

	Ongoing pregnancy *n* = 80	Miscarriage *n* = 23
Age	35.8 ± 3.6	36.3 ± 4.1
Endometrial thickness (mm)		
Day 1 of luteal suppport	10.2 ± 1.6	10.6 ± 2.0
Day 14 of luteal support	10.4 ± 2.3	10.7 ± 2.1
Pregnancy test day		
Progesterone (ng/mL)	14.7 ± 11.4*	9.6 ± 4.0**
Estradiol (pg/mL)	405.5 ± 192.9	351.5 ± 162.1
hCG (mIU/mL)	278.4 ± 182.0*	176.4 ± 97.2**

*Note*: Values are mean ± SD. * vs. **p < 0.05.

Abbreviations: hCG,human chorionic gonadotropin; LPS, luteal phase support.

**FIGURE 3:**
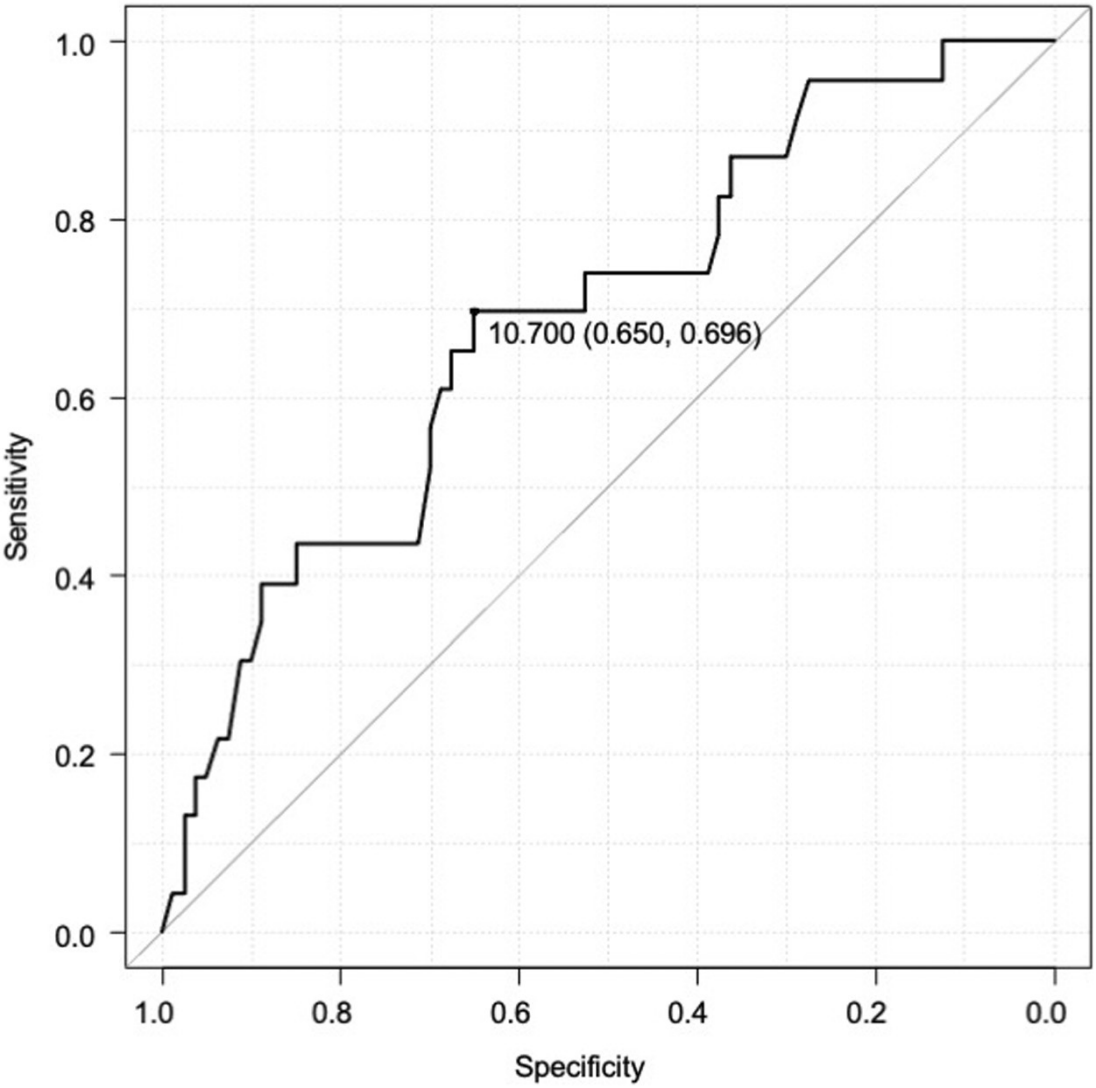
Receiver operatorating characteristic (ROC) curve of serum progesterone concentrations for evaluating the outcomes of assisted reproductive technology. The area under ROC curve 0.687 confidence interval 0.563‐0.811. ART, assisted reproductive technology. This ROC curve is a specification of EZR software.

Next, we determined the cut‐off value for serum P level on the ROC curve to evaluate the consequences of the pregnancy. The cut‐off value for the P level was 10.7 ng/mL, with an area under the curve of 0.69 (95% CI: 0.56–0.81) (Figure 3). Then, in the 103 hCG‐positive cases, we examined how the cut‐off value was related to the subsequent course of the pregnancy. A total of 61 cases (59.2%) showed P levels ≥10.7 ng/mL on day 14 of luteal support, whereas the remaining 42 cases (40.8%) had P levels <10.7 ng/mL. The backgrounds of the two groups are presented in Table 2. No significant differences were found between the two groups regarding age, body mass index, infertility period, causes of infertility, or anti‐Müllerian hormone level. Hormonal profiles and pregnancy outcomes in the two groups are shown in Table 3.

**TABLE 2:** Comparison of patients with serum progesterone levels no less than the cut off value or below it.

	P4 >= 10.7 group *n* = 61 (59.2%)	10.7 > P4 group *n* = 42 (40.8%)
Age (years)	35.6 ± 3.9	36.3 ± 3.4
Infertility period (years)	2.33	2.12
BMI (kg/m^2^)	21.1 ± 2.9	21.2 ± 2.9
Causes of infertility		
Tubal factor	0	1
Male factor	8	12
Endometriosis	6	1
Decreased ovarian reserve	3	2
Unexplained	43	21
Mixed	1	4
AMH (ng/mL)	4.6 ± 3.1	3.8 ± 2.4

*Note*: Values are mean ± SD.

Abbreviations: AMH, anti‐Müllerian hormone; BMI, body mass index.

**TABLE 3:** Hormonal profiles and clinical data of Study 1.

	P4 ≥ 10.7 group *n* = 61 (59.2%)	P4 < 10.7 group *n* = 42 (40.8%)
Pregnancy test day		
hCG (mIU/mL)	256.8 ± 176.8	253.8 ± 165.3
Estradiol (pg/mL)	384.1 ± 172.9	407.0 ± 206.9
Progesterone (ng/mL)	17.7 ± 11.8*	7.6 ± 2.0**
Fetal heart beat rate (%)	55 (90.2)*	32 (76.2)**
Ongoing pregnancy rate (%)	53 (86.9)*	27 (64.3)**
Miscarriage rate (%)	8 (13.1)*	15 (35.7)**

*Note*: Values are mean ± SD. * vs. **p < 0.05.

Abbreviation: hCG, human chorionic gonadotropin.

The average P level was 17.7 ng/mL for the non‐low P cases (≥10.7 ng/mL) and 7.6 ng/mL for the low P cases (<10.7 ng/mL). The average estradiol levels did not differ significantly between the two groups. There were 53 ongoing pregnancies at the end of 9 weeks of pregnancy (86.9%) and 55 fetal heartbeats positive cases (90.2%) in the non‐low P group. On the other hand, 27 cases (64.3%) were ongoing pregnancies at the end of 9 weeks of pregnancy, and 32 (76.2%) were fetal heartbeat‐positive cases in the low P group. The miscarriage rate in the low P group (35.7%) was approximately 2.7 times higher than that in the non‐low P group (13.1%). As for ongoing pregnancy, fetal heartbeat detection, and miscarriage rates, significant differences (*p* < 0.05) were found between the two groups, suggesting that the cut‐off value of serum P level showed a significant predictive value for the pregnancy outcomes.

### 3.2 Study 2

We then examined whether the combined administration of progestins with vaginal P in pregnant women with P levels being below the cut‐off value (<10.7 ng/mL) would reduce the miscarriage rate. We administered dydrogesterone 15 mg three times a day and intramuscular injection of P depot 125 mg weekly to 27 pregnant women, starting from day 14 of luteal support up to the completion of 9 weeks of pregnancy. As a result, there were 19 fetal heartbeat‐positive cases (70.4%) and 17 ongoing pregnancies at the end of 9 weeks of pregnancy (63.0%). Nevertheless, the outcomes were significantly inferior to those of the higher P group (≥10.7 ng/mL) in Study 1. Consequently, it seems that the higher miscarriage rate associated with low P levels cannot be rescued even if the additional progestins are administered after pregnancy is diagnosed.

### 3.3 Study 3

The results of Study 2 favor the idea that it is necessary to add progestins before the detection of hCG to reduce the miscarriage rate. Given this possibility, we attempted to co‐administer dydrogesterone from the beginning of luteal support with vaginal P. A total of 76 patients were administered both dydrogesterone and vaginal P. ART outcomes were compared between the non‐low P group (≥10.7 ng/mL) and the low P groups (<10.7 ng/mL) (Table 4). The two groups had no significant differences regarding age, body mass index, or anti‐Müllerian hormone level. The average P level was 15.4 ng/mL for the non‐low P cases (≥10.7 ng/mL) and 8.4 ng/mL for the low P cases (<10.7 ng/mL).

**TABLE 4:** Characteristics of patients and hormonal profiles and clinical data of Study 3.

	P4 ≥ 10.7 group *n* = 52 (68.4%)	P4 < 10.7 group *n* = 24 (31.6%)
Age (years)	36.3 ± 3.7	35.5 ± 3.9
BMI (kg/m^2^)	21.1 ± 2.9	21.1 ± 2.0
AMH (ng/mL)	4.3 ± 3.0	5.3 ± 5.2
Day 13 of luteal support		
Progesterone (ng/mL)	15.4 ± 4.9*	8.4 ± 1.3**
Estradiol (pg/mL)	381.0 ± 215.3	483.6 ± 306.1
Fetal heart beat rate (%)	47 (90.4)	22 (91.7)
Ongoing pregnancy rate (%)	44 (84.6)	20 (83.3)
Miscarriage rate (%)	8 (15.4)	4 (16.7)

*Note*: Values are mean ± SD.

Abbreviations: AMH, anti‐Müllerian hormone; BMI, body mass index.

The average estradiol levels did not differ significantly between the two groups. A fetal heartbeat was confirmed in 47 cases (90.4%) in the non‐low P group and 22 (91.7%) in the low P group. The numbers of ongoing pregnancies at the end of 9 weeks of gestation were 44 cases (84.6%) in the non‐low P group and 20 (83.3%) in the low P group. Thus, supplementation with dydrogesterone was found to improve pregnancy outcomes in women with low P levels to the same level as that in women with non‐low P levels.

## 4 DISCUSSION

FET in an artificially hormone‐treated cycle is the prevailing current of ART procedures. However, currently, no consensus guidelines regarding HRT regimens for FET have been formulated. Regarding luteal support, the type of progestin, optimal administration route, dosage, and duration vary depending on the medical facility. Under such circumstances, vaginal administration of P has become the mainstream luteal support.

Vaginal P inserts achieve higher endometrial concentrations, whereas serum P concentrations are relatively low compared with intramuscular injection of P.^3^ Although speculative, based on the notion that intrauterine P may be of prime importance for implantation and ensuing embryonic development, a transvaginal delivery system has been considered to be the preferred route for luteal support.^5^ As such, the vaginal P insert has been thought that its sole administration could be enough as luteal support by their properties to achieve high enough intrauterine concentrations of P, thus eliminating the need for measuring serum concentrations.^4^

In line with this claim, there have been reports describing that the outcomes of ART did not differ in terms of clinical pregnancy or live birth rates between vaginal and intramuscular P for luteal support in HRT‐FET cycles.^4^, ^12^, ^13^, ^14^ In addition to the efficacy of the vaginal inserts being equivalent to that of intramuscular injection of P, the satisfaction of vaginal insert seems superior to that of the intramuscular injection.^15^

With the rapid spread of the vaginal insert, there are increasing reports that luteal support with the vaginal P insert alone might results in a poor ongoing pregnancy rate in HRT‐FET cycles in some women who can benefit from the additional administration of progestins.^6^, ^9^ These observations could be best rationalized by the individual differences in the amount of absorbed P, as suggested that there was more than an order of magnitude variation in serum P levels even when the same dose of vaginal P was administered,^16^ as was further confirmed in this study (Figure 2). However, to date, no study has addressed the causal relationship between the serum P levels and the subsequent course of the pregnancy.

In this study, we demonstrated that the miscarriage rate was high in women who had low serum P levels with vaginal P alone as luteal support, and these women could be rescued by co‐administration of dydrogesterone together with vaginal P from the beginning, such that the ongoing pregnancy rate improved to a level comparable with that in women with non‐low serum P levels. These results demonstrated that, when using vaginal P alone, a significant proportion of women may lack P actions, resulting in a reduced ongoing pregnancy rate in HRT‐FET cycles. Perhaps insufficient P actions are reflected in low serum P levels; thus, progestin co‐administration seems beneficial to women with low serum P levels while receiving vaginal P.

This study consisted of three separate studies that were not conducted simultaneously. We could not conduct these three studies in parallel because the results of Study 1 inspired us to conduct studies 2 and 3 in this order. However, they were performed by the same doctors and the same embryologists in a single fertility clinic, and all ART procedures were basically identical except for the regimen of luteal support. Thus, we conclude that the results of these three studies are mutually comparable.

The present study showed that lower serum P levels, approximately 14 days after the start of luteal support in women conceived using HRT‐FET, were associated with poorer pregnancy outcomes. In the presence of a functional corpus luteum, the decrease in the serum P level may be the consequence of miscarriage, but in HRT‐FET pregnancies, the P level is not affected by embryonic developmental status. However, not all miscarriages associated with low P levels can be attributed to low P levels. A comparison of the miscarriage rates for the different P level groups (Table 3) indicated that the miscarriage rates were 13.1% for the non‐low P group and 35.7% for the low P group, whereas there were no differences between the two groups in terms of other conditions possibly related to the sustenance of pregnancy, as shown in Table 2. Based on these considerations, it can be estimated that low P might be responsible for more than 60% of miscarriages in the low P group, due to a shortage of P actions.

Although P is essential for pregnancy, the extent to which it acts is not the only determinant of ART outcomes. A closer look at the data of study 1 shows that 11 of the 103 patients with serum P levels of not <19.5 ng/mL were all ongoing pregnancies, suggesting that sufficient P may be highly favorable for the establishment and maintenance of pregnancy. However, there were a certain number of miscarriages when the P levels were <19.5 ng/mL even if the P levels were above the cut‐off value. Conversely, there were many ongoing pregnancies even when the P levels were below the cut‐off value (Figure 2). This implies that factors other than the P level are also involved in determining whether a pregnancy continues. When examining known factors associated with pregnancy outcomes, there were no differences in age, body mass index, endometrial thickness, and serum estradiol levels between the non‐low P and low P groups.^17^ All studies in the present study were conducted using identical ART equipment. In addition, the ART procedure employed in this study consisted of both basic and conventional methods. We did not utilize novel clinical tests or treatment techniques, whose efficacy had been explored but not yet established. Thus, regarding the result of this study, we could not identify any factors other than P that might influence ART outcomes. However, the present study examined serum P concentration, whereas the intrauterine P content may be directly related to the establishment and maintenance of pregnancy. To date, no apparent correlation between serum and intrauterine P concentrations has been confirmed.^3^ Additionally, P exerts its actions only after binding to its receptors. In this regard, the endometrium of women with endometriosis and polycystic ovary syndrome exhibits P resistance, presumably due to DNA methylation of the P receptor and aberrant P gene expression, respectively.^18^, ^19^ These are common diseases that account for many women undergoing ART, which could be one of the reasons why serum P levels alone cannot accurately predict ART outcomes. Despite these limitations, we focused on serum P concentration in the present study as the only practically measurable factor associated with P actions.

It is conceivable that if P levels are further reduced, even implantation could not occur. In this regard, no significant difference in the hCG positivity rate was noted between the women with the vaginal inserts alone and those supplemented with dydrogesterone (data not presented here). Therefore, implantation could be established even if a sufficient P level was not achieved with the vaginal insert alone. Thus, the question arises as to why supplementation with P after hCG detection in the lower P group did not contribute to a reduction in the miscarriage rate. Although highly speculative, peri‐implantation may be a P‐dependent critical period during which the pregnancy outcome is predetermined. However, if P levels are extremely low during embryo transfer, even implantation cannot occur.^20^

The data from the present study imply that luteal support with vaginal P alone seems inadequate in terms of P actions in a considerable proportion of women. As possible explanations for this, first, one may argue that there might be inter‐individual variability in the absorption of P through the vaginal wall.^16^, ^21^ Second, implantation and sustenance of pregnancy may require the actions of P present in peripheral blood vessels, as well as in the tissue fluid in the endometrium. In women with vaginal P, it increases P concentrations in the tissue fluid of the endometrium with certainty, but it may fail to increase P levels in the blood in many cases. It is possible that embryonic development is disrupted in such cases. This interpretation is in line with the association between serum P levels and ART performance, as observed in this study and other groups.^7^, ^8^

We have shown cases in which serum P levels are insufficient when only the vaginal P insert is solely administered. It may be argued that increasing the dose of the vaginal insert could solve this problem. However, the rate of miscarriage remained almost unchanged.^22^ The plausible reasons for this are as follows. First, the bioavailability of the transvaginal route is extremely low at 4%–8%, which means that even if a certain dose of vaginal P is administered, serum P concentrations are widely distributed. Secondly, even if the dose of vaginal preparation was increased from 25 to 100 mg, serum P concentration increased only by 35%; thus, the dosage of P and its serum concentrations were not proportional.^23^ It is likely that serum P concentrations level off at a given dose in the vaginal insert.

In the present study, the serum P cut‐off level of 10.7 ng/mL well‐discriminated ART outcomes. Similarly, Cédrin‐Durnerin et al. reported that the live birth rate differed significantly depending on whether the serum P level in FET was <10 ng/mL or ≥10 ng/mL.^16^ The cut‐off value of P mentioned above is limited to the cases where only vaginal P is administered. However, as long as intramuscular administration of P alone was applied as luteal support, the live birth rate was significantly different depending on whether the serum P level was <13.2 ng/mL or ≥13.2 ng/mL, and P levels was apparently higher thn that of vaginal P alone. Notably, the minimum serum P level associated with the success or failure of ART seems to be higher with intramuscular injection of P than that with vaginal P. Accordingly, it seems likely that the outcome of ART is not uniquely determined by the serum P concentration, whereas the intrauterine P level may play a pivotal role. It is interesting to speculate that in some cases where vaginal P is administered, intrauterine P levels may not be high enough for ART success, which might be reflected in lower serum P concentrations. Given this possibility, a low serum P level could be a surrogate marker for low intrauterine P levels.

Herein, we demonstrated that the combined administration of dydrogesterone and the vaginal insert increased the ongoing pregnancy rate in women conceived using FET in an HRT cycle. Intramuscular P injection is frequently used as luteal support to supplement the deficiency of P associated with the vaginal insert. In this study, however, we chose dydrogesterone because it does not contribute to the serum P concentration, which enabled us to evaluate the amount of P absorbed from the vaginal P preparation.^24^ This is why we could directly prove that the combined administration of dydrogesterone did not reduce the ongoing pregnancy rate even when the absorption of P from the vagina was insufficient. In addition, dydrogesterone is relatively inexpensive and considered to be reassuring when used during ART or during early pregnancy; therefore, it might be appropriate to administer it in all ART cases employing FET in an HRT cycle.^25^ In addition, oral preparations are painless in contrast to intramuscular injections. Similar findings conducted in Vietnam were reported by Vuong et al.^21^ However, their study did not measure serum P levels, whereas our study indicated that the co‐administration of dydrogesterone is particularly beneficial when vaginal P fails to achieve adequate serum P levels. If the pharmacokinetics study of the vaginal P in each woman is examined in advance before the start of HRT, dydrogesterone should be used only in women who are supposed to have lower P levels with the vaginal P. However, such a preliminary test is bothersome and time‐consuming. In addition, sufficient progestational actions should already act on the endometrium at the time of embryo transfer. Given that it takes 3 days for the blood concentration of dydrogesterone to reach a steady state after its administration, the start of luteal support appeared to be the right time to initiate dydrogesterone administration together with vaginal P.^26^ It might be argued that dydrogesterone alone can be used as luteal support without vaginal P. However, there are inter‐and intraindividual variations in serum dydrogesterone levels, with lower dydrogesterone levels being associated with a reduced ongoing pregnancy rate.^26^ Therefore, the combination of dydrogesterone with vaginal P seems to provide sufficient progestational actions as the sum of both in most cases, even if serum concentrations of both, or either, are low.

The present study had some potential limitations. First, this study had a retrospective and non‐randomized design. Moreover, three studies were not conducted in parallel. However, this study itself could not be justified without demonstrating that the miscarriage rate was associated with a decrease in the serum P level with vaginal P alone with our own study. Such circumstances inevitably caused a time lag between the periods in which each study was conducted. However, this study enabled us to gain insight into the inclusive issues related to the vaginal P inserts when used in HRT‐FET cycles. Exploring the effect of dydrogesterone in study 3 could have been performed in a randomized manner. However, we hesitated to do so because it was highly predictable, based on the results of the preceding studies, that using placebo would be disadvantage to participants.

Finally, although only micronized P soft capsules were used as the vaginal P in this study, the pharmacodynamics and bioavailability of the vaginal P may differ among currently available vaginal P preparations, which is an issue for future studies.^27^, ^28^

In conclusion, the single use of the vaginal P as luteal support results in a decrease in the ongoing pregnancy rate due to a lack of P actions in an appreciable proportion of women undergoing ART using HRT‐FET cycles. When using the vaginal P alone, serum P levels could be an indicator of insufficient P actions, which can be resolved by adding progestins in combination with vaginal P prior to embryo transfer.

## CONFLICT OF INTEREST STATEMENT

All the authors state explicitly that there are no conflicts of interest in connection with this article.

## ANIMAL STUDIES

Not applicable.

## HUMAN RIGHTS STATEMENTS AND INFORMED CONSENT

All procedures followed were in accordance with the ethical standards of the responsible committee on human experimentation (institute and national) and with the Helsinki Declaration of 1964 and its later amendment. Our IRB approved the study protocol and its consent form, and we obtained informed consent for this study from all participants.
